# Mr. Wilkinson, on Apoplexy, in Reply

**Published:** 1802-05

**Authors:** G. Wilkinson

**Affiliations:** Suuderland


### To Dr. BRADLEY.

SIR,

WHEN I volunteered my ſentiments on the diſpute between Mr. Crowfoot and Dr. Langſlow, which then appeared as a medical queſtion, and was ſubmitted to the free diſcuſſion of medical readers, I ſet out with that ſort of diffidence which every every candid inquirer after Truth knows how difficult it is to find her out, when encumbered with the fyftematic jargon and theoretical rubbifh with which fhe is fo oftentimes enveloped. I openly fubmitted what I had to fay, to the opinions and animadverfions of better judges than myfelf, more efpecially as *Apoplexy* muft be allowed to be a difeafe fatal in its nature, and ftill involved in doubt and obfcurity. It was to the *impartial* and *unbiased* part of profeffional readers that my Remarks were offered, and not to the parties concerned; for, as it will plainly appear that I have efpoufed the caufe of neither of them in particular, and profeffed myfelf to be of no party, I conceived myfelf at full liberty to canvafs the ftatements of thefe gentlemen with that ftrict juftice and impartiality that ought ever to actuate men of difpaffionate and liberal difpofitions. When individuals, differing in opinion, have confented to fubmit their difputes to the decifion of perfons disinterefted, as appears clearly from what has been offered by Mr. C. and Dr. L. to the inveftigation of the Republic of Medicine, or Medical Public, it might naturally be expected, particularly where fuch decifion has been given with candour, liberality, and a temper of mind divefted of perfonal animofity, that it fhould be acquiefced in; and where it has been enforced by the collateral or united opinions of others, equally uninfluenced by party motives, and wedded to no particular fyftem or doctrine, it is manifeftly obvious that a ftrict regard to the nature of the fubject in queftion, and an ardent defire of developing what cannot but be efteemed a doubtful, myfterious, and perplexing difeafe, muft be affuredly interefting, not only to thofe of the medical profeffion, but eminently beneficial to mankind. It is much to be regretted, after an appeal to the profeffion at large, with a view of collecting their fentiments on a doubtful fubject, that men, who by their acquirements, and the rank they hold in cultivated fociety, affect to profefs themfelves open to conviction, and yet degrade their underftandings by ftill adhering to errors, where fuch are by no means juftly tenable. If a decifion happens to fall out contrary to their vanity, not to mention the dubiety of their doctrines, and thefe advanced without mature and deliberate reflection, they unfortunately forget that while they tenacioufly hold them, it is the greateft of human weakneffes; and that they fhould remember, that inftead of conquering their own imperfections, they fuffer thofe very weakneffes or imperfections to conquer themfelves.

Can any thing be more ftrikingly contrafted to the conduct of Dr. Langflow, than that of the illuftrious and ingenious Dr. Baillie, a gentleman than whom no one more juftly occupies a more refpectable rank in his profeffion, and where it is difficult difficult to fay, whether he has not gained more lafting credit, and I may hope general approbation, in his recanting his error or miftake, than Dr. Hull, who fo fortunately and judicioufly explained it.* In this he has laudably imitated the example of Hippocrates, the prince of phyficians, who, by acknowledging he had miftaken a *suture of the cranium* for a *fissure*, has very juftly immortalized himfelf. Nor have there been wanting various other inftances of this fort, which cannot but be known to many of my readers verfed in Medical Biography, and whofe names it would be needlefs to point out, who fo far from confidering themfelves degraded by acknowledging their errors and imperfections, that they have deemed it as the greateft dignity accruing to human nature, to be made fenfible of the fallacy and uncertainty of that fort of fcientific knowledge, which can be but accounted as fpeculative.

I think I may venture to prefume, that not a few of my readers will agree with me, that it would have reflected no great difhonour on Dr. L. and tended greatly to have done away any miftaken prejudice that happened to prevail in the mind of Mr. C. towards him, if, after the fpeedy relief the patient experienced, he had barely admitted it as poffible that he might have been miftaken in his ideas of *extravasation*, or even *affusion*, particularly in this very cafe, which, at the beft, muft be allowed as doubtful. But, no! medical vanity was here too predominant, Dr. L. confiderered it as degrading to his confequence to condefcend even to allow an Apothecary or Surgeon to doubt; and when he found *extravasation*, which, according to his own definition in his original Statement, vol. vi. p. 561, of the Med. and Phys. Journal, implied *blood*, and that this feemed not quite tenable, he then reforts to ferum or lymph, by adopting thofe of *exudation* or *effusion*. Even his Explanation in vol. vii. of Med. Journal, p. 79, which is calculated to do away *extravasation*, which, as far as I can comprehend from both original Statements, appears to have been adopted by Dr. L. and which Mr. C. in his judgment, could not admit in the Cafe difputed, how eafy was it then to have terminated this difpute? Now it cannot be fatisfactorily and unequivocally refuted by Dr. L. that no fuch difeafe as the Nervous Apoplexy of Dr. Kirkland exifts, and which, according to the *erudite*, *ingenious*, and *liberal* difquifition of *Pyrrho*, appears to have been adopted or glanced at by feveral medical authors of refpectability, not even excepting the great Cullen himfelf,

* Medical Journal, No. xxxv. p. 32. No. xxxvi. p. 104.

himfelf,* Dr. L. can have no juft reafons or pretenfions to reject as *futile*, what to many, equally learned and ingenious, though not quite fo affuming, medical practitioners, appears an abfolute and even neceffary diftinction from that of the *sanguineous* and *serous* Apoplexy,† and which Dr. L. has, perhaps with more zeal than prudence, confidered as one and the fame, by admitting or allowing, that a fimilar mode of treatment might be applied in both cafes. I will readily admit Dr. L. is perfectly right where he quotes my having faid, that "the ingenious Doctor Kirkland confidered that fort of Apoplexy *to which Dr. L. alludes*, to be a difeafe *sui generis*." It was to the cafe in difpute that I meant to apply this remark; and this was advanced from Dr. K's work, with a view to prove that I believe Nervous Apoplexy does exift, although, at that time, I knew not of its being the opinion of Mr. Crowfoot. It is enough for me that Dr. L. has ranked me in the number of fuch *Ignoramuses* as the late worthy Dr. F. and Dr. K. together with Dr. G. and his antagonift Mr. C. and many others who may be of this opinion: But he ought to recollect, that although I have differed with him in his doctrines and opinions refpecting Apoplexy in general; I mean, in fuch cafes as have not terminated fatally, or where no *hemiplegia* fupervenes, it is obvious however that I have united with him in the propriety of the mode of treatment he adopted in the Cafe difputed, and even allowed it might be applied, with fome exceptions, generally: And as I have given no fanction to the ufe of emetics, not even in Nervous Apoplexy, I fhould think Mr. C. might, with as much propriety, have played me the *tune* of *St. Vitus's dance* as himfelf; but this I perceive he has not done, although in coinciding with that gentleman in his idea of *extravasation*, *exudation*, *&c.* I intended him no compliment: It was my decided opinion, and was fo prior to the difpute in queftion. But I forgive Dr. L. for his *wit*, though I confefs I prefer *wisdom* before it.

Dr. L. in his Anfwer to Mr. C's Statement, No. xxxiv. of Med. Journal, has given us the old Latin proverb, *Nemo mortalium omnibus horis sapit*; but whether he included its application to Mr. C. or himfelf, it is not for me to determine: certain however it is, that this *cap* would have fitted even the great *Solomon* himfelf. I cannot but own, however, in perufing the Doctor's Anfwer to my Remarks on his and Mr. C's Statements,

* Firft Lines, vol. iii.

† Med. and Phys. Journal, vol. vi. p. 561.

Statements, certain parts of which he has fingled out in detachments as the moft fuitable to his purpofe, and with a view of exercifing his farcaftic wit, I had much to do to reprefs my rifible mufcles; yet, when I reflected ferioufly on the importance and dignity of the fubject, the ftrict propriety and etiquette which ought to be adopted by men of liberal and enlightened principles, in difputations in which the benefit and happinefs of the human fpecies feem evidently implicated, I could not help feeling with regret the infinite pains and trouble Dr. L. muft have taken, to hunt out Mr. C's fuppofed literary affiftant, Dr. G. in the Norfolk and Suffolk villages,* and whom he was fo anxious to have dragged into this Controverfy, which he, Dr. G. it feems, perhaps more prudently than myfelf, carefully avoided, and where it appears Dr. L. muft have goffiped no inconfiderable part of his invaluable time; and this is the more aftonifhing, as he repeatedly complains of his being engaged by his *profeffional avocations*, which would induce me to imagine he had monopolized nearly the whole of the medical practice.

If I have declined entering into a farther contention or difpute with Dr. L. in his *ferious* comments on certain parts of my Remarks, he muft impute it to my averfion from traverfing repeatedly the fame paths; and as this has *already* been done by the elegant, enlightened, and candid *Pyrrho*, whofe difpaffionate and worthy to be imitated mode of difcuffion I wifh Dr. L. *for his own fake*, to adopt; and moreover, feeling myfelf *unwilling*, or, if it pleafe Dr. L. *unable* to anfwer him; for he has not fcrupled to affert, on account of Dr. G's filence to fome of his queftions, he, Dr. L. "trufts the public will naturally infer, that what he has there advanced is unanfwerable,"† he is at full liberty to impute to me the fame incapability. In any cafe where literary acumen, and a ftrict adherence to the fubject in queftion, combined with the utmoft accuracy of *perception*, is requifite, that man muft be regarded as an object of peculiar commifferation who, inftead of bearing in mind thefe important requifites, *indulges* in vanity, and exhibits to the world all the flatulency of ill-concocted erudition. This Reply to Doctor Langflow might, prior to this, have been written and fent to the Medical and Phyfical Journal, had not my *profeffional avocations*, *lectures*, and certain papers *preparing* for the prefs, prevented me; neverthelefs, I am not without hopes,

* Med. and Phys. Journal, No. xxxvii. p. 274, et feq.
† Med. and Phys. Journal, No. xxxvii.

hopes, *the moment Dr. L. has a little leisure*, he will certainly favour us with his promifed work on Apoplexy.

I am, Sir, yours, &c.

*Suuderland, April* 10, 1802. G. WILKINSON.

P. S. In reply to Mr. Atkinfon's polite query in No. 37, Med. Journal, refpecting frothing at the mouth, Mr. W. admits he is *incorrect*, and that this is more *decisively characteristic of Epilepsy*. Spafms of the mufcular fyftem, however flight, may be confidered as fuch, in order to diftinguifh them from convulfions; Dr. Baillie allows of *convulsive motions*, fee Appendix to Morbid Anatomy; and I think Dr. Cullen the fame, fee Firft Lines, vol. iii. Even in the Cafe difputed, it appears from Mr. Crowfoot's Statement, that the patient was *convulsed*, fee Med. Journal, vol. vi. p. 549; Mr. W. neverthelefs, affures Mr. A. that in the cafe of the man, aged 63, defcribed by him in vol. vii. p. 145, the mufcular fyftem was ftrongly agitated, i. e. comparatively. Mr. W. can fee no very material reafons why thefe fymptoms fhould be *decisively* characteriftic of Epilepfy; all allow thefe two difeafes to be fo nearly allied as not always to be correctly afcertained, but with the exception that one ufually attacks *youth*, and the other perfons advanced in life.

